# Prevalence and Cardiovascular Associations of Diabetic Retinopathy and Maculopathy: Results from the Gutenberg Health Study

**DOI:** 10.1371/journal.pone.0127188

**Published:** 2015-06-15

**Authors:** Philipp Raum, Julia Lamparter, Katharina A. Ponto, Tunde Peto, René Hoehn, Andreas Schulz, Astrid Schneider, Philipp S. Wild, Norbert Pfeiffer, Alireza Mirshahi

**Affiliations:** 1 Department of Ophthalmology, University Medical Center, Mainz, Germany; 2 NIHR Biomedical Research Center at Moorfields Eye Hospital and UCL Institute of Ophthalmology, London, United Kingdom; 3 Biomedical statistics, University Medical Center, Mainz, Germany; 4 Center for thrombosis & haemostasis (CTH), University Medical Center, Mainz, Germany; 5 Department of Medicine II, University Medical Center, Mainz, Germany; 6 German Center for Heart and Circulations System Research (DZHK), University Medical Center, Mainz, Germany; 7 Dardenne Eye Hospital, Bonn, Germany; Schepens Eye Research Institute/Massachusetts Eye and Ear, Department of Ophthalmology, Harvard Medical School, Boston, MA, UNITED STATES

## Abstract

**Objective:**

Diabetic retinopathy (DR) is the leading cause of blindness in people of working age. The purpose of this paper is to report the prevalence and cardiovascular associations of diabetic retinopathy and maculopathy (DMac) in Germany.

**Research Design and Methods:**

The Gutenberg Health Study (GHS) is a population-based study with 15,010 participants aged between 35 at 74 years from the city of Mainz and the district of Mainz-Bingen. We determined the weighted prevalence of DR and DMac by assessing fundus photographs of persons with diabetes from the GHS data base. Diabetes was defined as HbA1c ≥ 6.5%, known diagnosis diabetes mellitus or known diabetes medication. Furthermore, we analysed the association between DR and cardiovascular risk factors and diseases.

**Results:**

Overall, 7.5% (1,124/15,010) of the GHS cohort had diabetes. Of these, 27.7% were unaware of their disease and thus were newly diagnosed by their participation in the GHS. The prevalence of DR and DMac was 21.7% and 2.3%, respectively among patients with diabetes. Vision-threatening disease was present in 5% of the diabetic cohort. In the multivariable analysis DR (all types) was associated with age (Odds Ratio [95% confidence interval]: 0.97 [0.955–0.992]; p = 0.006) arterial hypertension (1.90 [1.190–3.044]; *p* = 0.0072) and vision-threatening DR with obesity (3.29 [1.504–7.206]; p = 0.0029). DR (all stages) and vision-threatening DR were associated with duration of diabetes (1.09 [1.068–1.114]; p<0.0001 and 1.18 [1.137–1.222]; p<0.0001, respectively).

**Conclusions:**

Our calculations suggest that more than a quarter-million persons have vision-threatening diabetic retinal disease in Germany. Prevalence of DR was lower in the GHS compared to East-Asian studies. Associations were found with age, arterial hypertension, obesity, and duration of diabetes mellitus.

## Introduction

Diabetic retinal and macular diseases are the main cause of legal blindness in adults aged between 20 and 74 years in industrial nations [1,2]. Diabetic retinopathy (DR) is a microangiopathic complication of diabetes mellitus (DM) being associated with cardiovascular risk factors [3,4]. It has been shown that neuronal and glial alterations may precede the overt vascular changes that characterize DR [5–10].

The prevalence of DR varies considerably according to geography, patients' ethnicity, and their way of life [4]. To date, there are no population-based data on the prevalence of diabetic retinal and macular pathologies in Germany. From a healthcare standpoint, data on the prevalence and incidence of diabetic retinopathy are an essential basis for the future planning of healthcare provision.

The Gutenberg Health Study (GHS) is a prospective, population-based interdisciplinary study being conducted by University Medical Centre in the city of Mainz and the Bingen-Mainz region; it has 15,010 participants aged between 35 and 74 years [11–13].

The aim of our investigation was to describe the prevalence of diabetic retinopathy and maculopathy in a large German cohort and to analyse their association with cardiovascular risk factors and diseases.

## Methods

### Study participants

We recruited 15,010 participants aged between 35 and 74 years living in the city of Mainz or the region of Mainz-Bingen for the *Gutenberg Gesundheitsstudie* (English: Gutenberg Health Study, or GHS) (Table 1). The GHS is a prospective, monocentric, population-based cohort study designed to examine diseases of the eye, the cardiovascular system, the psyche and the immune system [11,12]. Main objectives of the ophthalmological branch of the study are to convey the prevalence and incidence of common ophthalmological risk factors and diseases as well as to investigate interdisciplinary correlations and their genetic background. The random sample is stratified 1:1 for the cohort's gender and residence (urban versus rural), and in equal proportions across four age decades. The GHS was approved by the Ethics committee of Rhineland-Palatinate. All persons gave their written informed consent prior to their inclusion in the study.

**Table 1:** Gutenberg Health Study cohort.

	Total; n = 15,010	Male; n = 7,584	Female; n = 7,426
Age (mean ± SD; in years)	55.0 (±11.1)	55.3 (±11.1)	54.8 (±11.1)
BMI(mean ± SD; kg/m^2^)	27.4 (±5.0)	27.9 (±4.3)	26.9 (±5.6)
Hypertension (%)	49.8	54.6	44.8
Diabetes mellitus (%)	7.5	9.5	5.5
Smoking (%)	54.2	61.6	46.6
Dyslipidaemia	29.5	36.7	22.2
History of myocardial infarction (%)	3.0	4.5	1.4
Obesity (%)	25.2	26.3	24.1
HbA1c (%)	5.5	5.5	5.5

We defined a study participant as diabetic if he or she fulfilled at least one of these criteria:

- diabetes mellitus diagnosed by a physician
- known therapy for diabetes mellitus (dietetic, oral, or insulin)
- HbA1c ≥6,5%

We had access to photographic images of the fundus and complete examination findings of 1,045 of the 1,124 (93.0%) diabetics in the study cohort. Of those, we had to exclude 102 (9.1%) patients due to images of inadequate quality, leaving 943 assessable fundus images of the diabetics.

### Fundus images, grading and cardiovascular risk factors

The fundus images were taken with a non-mydriatic fundus camera (Visucam PRO NM, Carl Zeiss AG, Jena, Germany) in a darkened room and with the pupil’s natural width. Three photographs were taken of each eye: at 30° and 45° centred on the optic nerve, and at 30° centred on the macula.

These images were all evaluated at the Reading Centre at Moorfields Eye Hospital in London, UK at a specifically-designed work station by two certified graders (PR, JL). The fundus images were the basis upon which we diagnosed diabetic retinopathy or not and if so, its stage. The stage of DR was determined according to criteria applied in the Early Treatment Diabetic Retinopathy Study [14] as mild, moderate, severe non-proliferative, or as proliferative diabetic retinopathy. We also assessed whether a diabetic maculopathy was present. Table 2 illustrates the simplified ETDRS criteria for the stages of DR and DMac. In the presence of

- severe non-proliferative diabetic retinopathy and/or
- proliferative diabetic retinopathy and/or
- diabetic maculopathy,

they were graded in line with the epidemiological literature as a vision-threatening disease [2]. If the two eyes yielded discordant diagnoses, we referred to the eye presenting the most advanced stage for that patient's assessment.

**Table 2:** Grading criteria of diabetic retinopathy (DR) and maculopathy (simplified EDTRS criteria [14].

Stage	Definition
Mild non-proliferative DR	One of the following: microaneurysm, retinal haemorrhage, hard / soft exudate, venous beading
Moderate non-proliferative DR	Combination of two characteristics of mild non-proliferative DR
Severe non-proliferative DR	Microaneurysm / retinal haemorrhage in 4 quadrants, venous beading in 2 quadrants, intraretinal microvascular anomaly in 1 quadrant
Proliferative DR	Visible laser scars, neovascularisation at disc or elsewhere, subhyaloidale haemorrhage, vitreous haemorrhage
Diabetic maculopathy	Exudate, oedema, haemorrhage, microaneurysm adjacent to the fovea

The baseline examinations of the GHS study participants incorporated classic cardiovascular risk factors, namely:

- arterial hypertension (blood pressure syst./diast. ≥140/90 as the mean from several measurements, a physician's diagnosis, or blood-pressure medications being taken),
- nicotine abuse (yes: current smoker, no: never, or ex-smoker)
- overweight (BMI ≥ 30kg/m^2^)
- family disposition for myocardial infarction (coronary heart disease among the patient's immediate relatives before age 60 (m) or 65 (w))
- dyslipidaemia (presence or a physician-verified lipid-metabolism disorder (self-reported), LDL/HDL ratio).

Taking the participants' histories we also investigated whether gender, age, presence of peripheral arterial occlusive disease, atrial fibrillation, chronic heart or renal insufficiency, coronary heart disease, and previous stroke or heart attack were associated with the diagnosis of diabetic retinopathy.

For quality-control purposes we had the fundus images of 218 age and gender-matched controls assessed who did not have diabetes.

### Statistical Analysis

Prevalences are given as relative numbers in percent. Continuous variables are given as the mean and standard deviation, or as the median with 25–75 percentiles

We determined the weighted, gender- and age-specific prevalences of diabetic retinopathy and maculopathy as well as of any vision-threatening disease. As our study participants were stratified across gender and four age decades in equal proportions, they were weighted according to their actual age and gender distribution in their region. Weighting was based on census data as of 31.12.2009, coinciding approximately with the midpoint in our recruitment phase. As the distribution of missing values was not age- and gender-independent, weighting was done after having removed them.

Via the logistic regression model we determined whether the presence of DR was associated with age and sex and with the cardiovascular diseases and risk factors, as well as with the duration of diabetes in these individuals. We also examined whether the presence of vision-threatening disease stage was associated with those systemic diseases. A univariable binary logistic regression was performed to first show a crude odds ratio. A confounder adjusted odds ratio was calculated in a multivariable binary logistic regression model

## Results

### Study participants

In the GHS, 7.5% (1,124/15,010) of the study participants were by definition suffering from diabetes mellitus (9.5% males vs. 5.5% females). The vast majority of the diabetics (79.6%) had Type-II diabetes, 6.0% Type-I diabetes. We diagnosed gestational diabetes in 0.7% and diabetes after pancreatitis in 0.3%. The clinical classification was unclear in 13%.

Both fundus images and complete baseline-examination findings were available from 1,045 0f 1,124 (93.0%) of the diabetics.

Of the diabetics, 72.3% knew they had the disease. Most of them (42.8%) were taking an oral anti-diabetic, 17.0% required insulin, and 10.2% were taking both on combination therapy. Therapy was diet-based in 1.2%, while 1.1% were not on any therapy at the examination time point. In the questionnaire, 27.7% stated they did not have diabetes or were not taking any anti-diabetic medication (screening-detected disease).

Those in the study cohort with diabetes mellitus had an excessive Body Mass Index, BMI (Q1-/Q3-Median): 30.7 kg/m^2^ (27.3/34.3). The cohort's diabetics presented a median HbA1c value measuring 6.80% (6.40/7.30).

### Prevalences

The total weighted prevalence of any DR in the group of diabetics from the city of Mainz and Mainz-Bingen region was 21.7%. Of the females, 22.6% and 21.1% of the males had DR.

Tables 3 and 4 illustrate the prevalences according to disease stage and age.

The weighted prevalence of DMac was 2.3% in the group containing all the diabetics; 2.0% of the males versus 2.9% of the female diabetics presented diabetic ocular anomalies in the central retina.

**Table 3:** Weighted prevalence of diabetic retinopathy (DR).

Stage	Prevalence
No DR	78.3%
Mild non-proliferative DR	14.9%
Moderate non-proliferative DR	2.3%
Severe non-proliferative DR	2.4%
Proliferative DR	2.0%

**Table 4:** Weighted prevalence of diabetic retinopathy in different age decades.

Age decade (years)	Prevalence
35–44	28.2%
45–54	24.6%
55–64	19.6%
65–74	20.9%

The weighted prevalence of vision-threatening disease was 5.0%: 4.9% in the males and 5.1% in the females. Table 5 shows the prevalences of vision-threatening disease according to the patients' decades of age.

**Table 5:** Weighted prevalence of vision-threatening disease in different age decades.

Age decade (years)	Prevalence
35–44	4.9%
45–54	4.8%
55–64	4.5%
65–74	5.4%

### Associations

In the univariable analyses (Table 6) we identified an association of presence of DR (at any stage) with arterial hypertension (Odds Ratio [95% confidence interval]: 1.79 [1.174–2.719]; p = 0.0068) and duration of diabetes (1.08; 1.068–1.106; p<0.001). The remaining cardiovascular risk factors revealed no association with DR, neither with age nor with gender. The presence of revealed an association with duration of diabetes (1.16; 1.122–1.196; p<0001). We did, however, note a positive correlation between the stage of DR and HbA1c- and BMI values (Table 7). In the multivariable binary logistic regression analyses (Table 8) presence of DR (at any stage) was associated with age (0.97; 0.955–0.992; p = 0.0062), arterial hypertension (1.90; 1.190–3.044; p = 0.0072), and with diabetes duration (1.09; 1.068–1.114; p<0.0001), whereas presence of vision-threatening DR was associated with obesity (3.29; 1.504–7.206; p = 0.0029) and with duration of diabetes (1.18; 1.137–1.222; p<0.0001).

**Table 6:** Univariable analysis: Logistic regression model.

	Diabetic retinopathy (DR) overall			Vision-threatening DR		
	OR	95% CI	p-value	OR	95% CI	p-value
Sex (reference: women)	1.08	0.793–1.465	0.63	1.06	0.593–1.894	0.84
Age	0.99	0.973–1.007	0.24	1.01	0.972–1.040	0.76
Familiy history of myocardial infarcion	1.16	0.802–1.673	0.43	0.76	0.352–1.651	0.49
Obesity	1.25	0.920–1.693	0.15	1.77	0.966–3.256	0.065
Arterial hypertension	**1.79**	**1.174–2.719**	**0.0068**	1.37	0.633–1.966	0.42
Dyslipidemia	1.13	0.835–1.523	0.43	0.98	0.553–1.725	0.93
Smoking	1.09	0.732–1.634	0.66	0.85	0.377–1.934	0.70
Diabetes duration	**1.08**	**1.063–1.106**	**< 0.0001**	**1.16**	**1.122–1.196**	**< 0.0001**

Odds ratios refer to overall diabetic retinopathy (DR) or to vision-threatening (DR). Results with p<0.05 in bold letters.

**Table 7:** Body mass index (BMI) in different stages of diabetic retinopathy.

Stage	BMI (kg/m^2^)	HbA1c (%)
No DR	28.5 (25.5/32.3)	6.00 (5.80/6.60)
Mild non-proliferative DR	29.2 (26.2/32.9)	6.30 (5.80/6.90)
Moderate non-proliferative DR	31.4(28.3/35.4)	7.20(6.32/7.88)
Severe non-proliferative DR	32.5(25.6/37.1)	8.05(7.30/8.62)
Proliferative DR	35.0(28.9/40.5)	7.70(6.93/9.37)

**Table 8:** Multivariable analysis: Logistic regression model. Odds ratios (OR) refer to overall diabetic retinopathy (DR) or to vision-threatening (DR).

	Diabetic retinopathy (DR) overall			Vision-threatening DR		
	OR	95% CI	p-value	OR	95% CI	p-value
Sex (reference: women)	1.11	0.797–1.536	0.55	0.95	0.482–1.858	0.87
Age	**0.97**	**0.955–0.992**	**0.0062**	0.98	0.941–1.021	0.33
Familiy history of myocardial infarcion	0.97	0.656–1.439	0.89	0.48	0.197–1.149	0.099
Obesity	1.26	0.901–1.747	0.18	**3.29**	**1.504–7.206**	**0.0029**
Arterial hypertension	**1.90**	**1.190–3.044**	**0.0072**	1.64	0.579–4.660	0.35
Dyslipidemia	1.05	0.762–1.441	0.78	0.90	0.465–1.737	0.75
Smoking	1.29	0.828–2.019	0.26	0.99	0.354–2.782	0.99
Diabetes duration	**1.09**	**1.068–1.114**	**< 0.0001**	**1.18**	**1.137–1.222**	**< 0.0001**

Results with p<0.05 in bold letters.

The quality-control examinations revealed no vision-threatening disease in any control patient however, somewhat over 10% had retinal changes that would have been assessed as a mild stage of DR.

## Discussion

The GHS currently has the largest population-based study cohort in Germany to have ever been investigated in terms of the prevalence of diabetic retinopathy and maculopathy. Our study is thus delivering findings from the European country in with the largest population. We were astounded to learn that over a quarter of these individuals were unaware they had diabetes, revealing a high number of unreported cases of diabetes in Germany. These individuals cannot benefit from secondary prevention measures (which would help avoid the comorbidities associated with diabetes) because they are unknown of their disease. This does not just have consequences for the affected individual who—in a worse-case scenario—may go blind because of retinopathy. It could also create a heavy socioeconomic burden on the state due to high costs for disability.

### Prevalence of diabetic retinopathy

About a fifth of the diabetics aged between 35 and 74 in this study were diagnosed with DR, with about a third of them presenting an advanced stage of the disease. Approximately a third of those affected were in a moderate, severe or even proliferative stage of the disease. Of the diabetics in the study cohort, 5% were threatened by vision loss. The GHS data therefore enables us to state that a quarter of a million persons in Germany (N = 257.600) are at risk of losing vision secondary to diabetes mellitus (0.32% of 80.5 million inhabitants) [15].

One of the key results of the GHS is our disclosure of a distressingly large number of unreported cases of diabetes mellitus in Germany and the relatively high prevalence of this vision-threatening disease in an industrial nation. Both results are economically and socially relevant, and will possibly incur drastic increases in healthcare costs. We report certain cardiovascular risk factors being associated with diabetic retinopathy, namely arterial hypertension and history of a myocardial infarction.

Table 9 summarises the prevalences of diabetic retinal and maculopathy and vision-threatening disease in the GHS in comparison with other population-based studies. In Europe data from large population-based cohorts with diabetes are available from the Netherlands [16] and Norway [17]. The reported prevalences were somewhat higher than those in the GHS. A study from 2006 to 2008 [2] delivered data from the USA as a western but non-European industrial nation. The prevalence of DR was 28.5% and thus a bit higher than in the GHS cohort. Diabetic maculopathy and vision-threatening disease were reported at 2.7% and 4.4% respectively being comparable to our results. Other more recent investigations on the epidemiology of diabetic retinopathy stem largely from Asian countries. Those studies from China [3,18] and Singapore [19] revealed prevalences much higher than ours. The lowest reported prevalence data are reported from the Indian (17.6%) and (19%) Arabic subcontinent [20,21].

**Table 9:** Prevalences of diabetic retinopathy and maculopathy in other population-based studies.

Study	Country	N	Prevalence of diabetic retinopathy (%)	Prevalence of diabetic maculopathy (%)	Vision-threatening disease (%)
Gutenberg Health Study	Germany	943	21.7	2.3	5.0
Tromsø Eye Study [17]	Norway	514	26.8	3.9	Not applicable (visual acuity <0.3: 1.6%)
Prevalence of diabetic retinopathy in the United States [2]	USA	1006	28.5	2.7	4.4
Beijing Eye Study [3]	China	362	27.9	4.0	~ 20%
Singapore Malay Eye Study [19]	Singapore	757	35.0	5.7	9.0
Handan Eye Study [18]	China	368	43.1	no data available	no data available
Blue Mountains Eye Study [40]	Australia	252	29.0	no data available	no data available
Chennai Urban Rural Epidemiology Study [21]	India	1715	17.6	5.0	no data available
UAE Cross-sectional Survey [20]	United Arab Emirates	513	19.0	no data available	no data available

The diversity of the data on prevalence can be attributed to typical influencing factors behind epidemiological studies. These are essentially ethnic background, environmental effects, genetic factors, and life style, as well as a study's methodology, i.e., its diagnostic criteria. In this study we based our DM diagnosis on the HbA1c value in those individuals whose disease was not previously known: in this case, diabetes was defined as an HbA1c value of 6.5% or higher [22]. The application of other disease definitions based on fasting glucose values or an oral glucose-tolerance test may have revealed more or fewer diabetics in the GHS cohort and thus other prevalences. We based our disease definition on the HbA1c value because it is easy to assess, the assays are standardised, and because it is a widely-used parameter for diagnosing DM. Malkani and Mordes report in an overview article that one will identify fewer persons with diabetes mellitus using the HbA1c criteria compared to the conventional definition by blood glucose test [23]. However, there appears to be ethnic differences as Jorgensen et al report in a comparative study between Danish and Inuit [24]. Similarly results of the German KORA study show that there are certain differences in the number of diabetics when using the two different methods of diagnosis [25].

While the frequency of DM is influenced by the diagnostic criteria applied, technical and therapeutic factors play a role when determining the prevalence of diabetic retinal and macular diseases, as do diverse examination conditions. Optic coherence tomography (OCT) is a technical development that has revolutionised ophthalmological diagnostics, especially for macular diseases. OCT enables us to image the central retina in layers and to visualise and assess macular oedema [26]. We did not have access to OCT images from our study cohort and thus cannot make any statements as to the presence or severity of diabetic macular oedema.

One possible reason for the underestimation of DR‘s prevalence revealed by our study is that we did not carry out medical pupil dilation. Nevertheless, our study methodology is comparable to other epidemiological investigations done under similar conditions, such as that by Zhang et al. [2] on the prevalence of DR in the United States. A recent review article shows that 14 out of 35 studies on diabetic retinopathy have not applied pupil dilation by medication [4]. The relatively high percentage (>10%) of mild DR diagnosed in our control group can be attributed to the definition of DR (already present when a microaneurysm is visible), to image quality, and to other retinal microangiopathies. Yet not one patient was identified in the control group in a vision-threatening stage of the disease.

Moreover, it is conceivable that success in treating early stages of the disease will result in less severe manifestations of diabetic maculopathy in the future. For example, the recent introduction of intravitreal anti-VEGF therapy has improved the chances of preserving residual vision in patients with certain forms of diabetic maculopathy [27,28], and a recently-approved intravitreally-administered cortisone compound (fluocinolone acetonide) has delivered promising results even in patients with therapy-resistant diabetic macular oedema [29,30]. Further epidemiological studies are needed to assess the impact of new treatment options on prevention of vision loss secondary to diabetes mellitus.

### Association between diabetic retinopathy and cardiovascular risk factors

There is still much work to be done in terms of prevention, patient care and public education because of the high rate of individuals unaware of their disease and because of DR’s association with other cardiovascular illnesses [31]. In our study we discovered an association between the presence of DR and arterial hypertension and with the duration of diabetes in these persons. Furthermore, vision-threatening DR was associated with obesity. There is evidence of an association between DR and arterial hypertension [2,3,19] and dyslipidaemia [3,19]. Arterial hypertension plays a highly relevant role in the progression of retinopathy: the United Kingdom Prospective Diabetes Study demonstrated that the risk of DR progression can be reduced by about 34%, and the risk of a significant loss of visual acuity by even 47% in conjunction with a target blood pressure under 150/85 mmHg compared with one under 180/105 mmHg [32]. It has been shown that obesity may possibly play an important role in endothelial dysfunction involved in the pathogenesis of DR [33]. The association we observed between DR and arterial hypertension and obesity reinforces the acknowledged association between DR or diabetes, and (risk factors for) arteriosclerosis [34]. Altered cardiometabolic risk profiles, and elevated blood pressure in particular, have shown to be significantly associated with chronic complications in overweight and obese patients with type 2 diabetes [35]. This is in line with the association found between vision-threatening DR and obesity in persons with diabetes in the GHS. Furthermore, the association between obesity and vision-threatening DR might be explained by the fact that macular thickness and volume seem to be directly associated with higher serum lipid levels [36].

We were unable to address the Tromsø Eye Study [17] team‘s association between DR and microalbuminuria because the GHS examinations did not include testing that parameter. The GHS failed to detect any association between chronic renal insufficiency and DR.

The GHS’s responder rate was 60.2%. The extent to which potential immobility, inability or the poor vision of patients with diabetes may have prevented study participation (and thus influenced the prevalences we observed) cannot be evaluated (selection bias), as we have no access to such information.

Another limitation of the present study merits consideration: There might have been bias as this cohort potentially included some cases of prediabetic subjects who were treated with oral antidiabetic drugs (e. g., Metformin). It has been shown that Metformin decreases the rate of conversion from prediabetes to diabetes [37]. Therefore, it might be that—according to the above-mentioned definition- we mis-classified some prediabetics to have a manifest DM. Nevertheless, as this prophylactic therapy is not recommended according to the Guidelines of the German Diabetes Association [38], we suppose that the number of prediabetic persons treated with off-label oral antidiabetic drugs was rather low. The results on the prevalence of DR in prediabetes have been published previously [39].

### Future perspectives

The prospective GHS is currently (since April 2012) in its 5-year follow-up phase during which study participants are being re-examined. We anticipate being able to deliver initial data on the incidence of diabetes mellitus and of DR later this year. Furthermore, we will be able to provide more detailed clinical information on any macular involvement thanks to OCT imaging being introduced to the GHS in the follow-up phase.
