# Correction: Prevalence and Cardiovascular Associations of Diabetic Retinopathy and Maculopathy: Results from the Gutenberg Health Study

**DOI:** 10.1371/journal.pone.0139527

**Published:** 2015-09-25

**Authors:** Philipp Raum, Julia Lamparter, Katharina A. Ponto, Tunde Peto, René Hoehn, Andreas Schulz, Astrid Schneider, Philipp S. Wild, Norbert Pfeiffer, Alireza Mirshahi

There are errors in the first sentence of the “Conclusions” subsection of the Abstract. The correct sentence is: Our calculations suggest that approximately 142 000 persons aged between 35 and 74 years have vision threatening diabetic retinal disease in Germany.

There are errors in the fourth sentence of the “Prevalence of diabetic retinopathy” subsection of the Discussion. The correct sentence is: The GHS data therefore enables us to state that approximately 142 300 persons in Germany aged between 35 and 74 year are at risk of losing vision secondary to diabetes mellitus (0.33% of 43 123 995 inhabitants) [15].

## References

[1] Raum P, Lamparter J, Ponto KA, Peto T, Hoehn R, Schulz A, et al (2015) Prevalence and Cardiovascular Associations of Diabetic Retinopathy and Maculopathy: Results from the Gutenberg Health Study. PLoS ONE 10(6): e0127188 doi:10.1371/journal.pone.0127188 26075604 10.1371/journal.pone.0127188 PMC4468098

